# An integrated complete-genome sequencing and systems biology approach to predict antimicrobial resistance genes in the virulent bacterial strains of *Moraxella catarrhalis*

**DOI:** 10.1093/bfgp/elaf027

**Published:** 2026-02-02

**Authors:** Sadia Afrin Bristy, Md Arju Hossain, Md Imran Hasan, S M Hasan Mahmud, Mohammad Ali Moni, Md Habibur Rahman

**Keywords:** *Moraxella catarrhalis*, clustering analysis, functional enrichment analysis, antimicrobial resistance system, AMR genes, gene ontology

## Abstract

*Moraxella catarrhalis* is a symbiotic as well as mucosal infection-causing bacterium unique to humans. Currently, it is considered as one of the leading factors of acute middle ear infection in children. As *M. catarrhalis* is resistant to multiple drugs, the treatment is unsuccessful; therefore, innovative and forward-thinking approaches are required to combat the problem of antimicrobial resistance (AMR). To better comprehend the numerous processes that lead to antibiotic resistance in *M. catarrhalis*, we have adopted a computational method in this study. From the NCBI-Genome database, we investigated 12 strains of *M. catarrhalis*. We explored the interaction network comprising 74 antimicrobial-resistant genes found by analyzing *M. catarrhalis* bacterial strains. Moreover, to elucidate the molecular mechanism of the AMR system, clustering and the functional enrichment analysis were assessed employing AMR gene interactions networks. According to the findings of our assessment, the majority of the genes in the network were involved in antibiotic inactivation; antibiotic target replacement, alteration and antibiotic efflux pump processes. Additionally, *rpoB, atpA, fusA, groEL* and *rpoL* have the highest frequency of relevant interactors in the interaction network and are therefore regarded as the hub nodes. These hub genes only reflects their centrality in cellular function, rather than direct or selective targets for antimicrobial development without reservation. Finally, we believe that our findings could be useful to advance knowledge of the AMR system present in *M. catarrhalis* via a series of phenotypic assays including MIC testing, and gene expression analysis (RT-qPCR) to confirm the functional expression of AMR genes.

## Introduction

**Figure 1:**
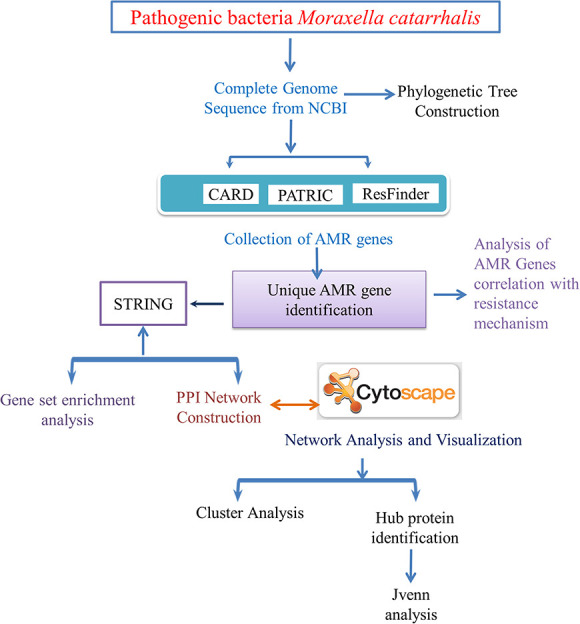
This workflow illustrates the whole exploration of how we predicted antibiotic resistance in *M. catarrhalis* by applying multiple computational techniques, depending on complete-genome sequencing and systems biology approaches.

Antimicrobial resistance (AMR) in pathogenic bacterial strains is currently a serious problem causing a lot of death and suffering to mankind around the globe. It has a negative impact on diagnostic, therapeutic and financial consequences, with implications varying from a patient’s failure to retaliate to treatment. Moreover, the rising incidence of multidrug-resistant (MDR) bacterial pathogens causing clinical and community-acquired diseases is restricting antibiotic treatment choices [1]. There are several pathogens found in the intensive care unit that can develop antibiotic resistance, but gram-negative strains of bacteria are the most prone to developing barriers to various kinds of antibiotics [2]. The most frequent methods of resistance to β-lactam in gram-negative bacteria are antimicrobials obliteration *via* beta-lactamases; insulative properties, which include the shutdown of gene encoding channels in the bacterial cell membrane; and antibiotic deformation by efflux pumps [3]. To better understand the numerous antibiotic resistance systems that lead to AMR in *Moraxella catarrhalis*, we employed a computational method in our current study (Figure 1).

*Moraxella catarrhalis* is a gram-negative, gamma-proteobacterium and ox*ygenated ubiquitous* bacteria which generates acute otitis media (AOM) in youngsters and reduced respiratory tract problems in adults, putting a strain on medical infrastructures across the world [4]. Previously, this microorganism was referred to as *Neisseria catarrhalis* or *Micrococcus catarrhalis* [5]. It is commonly encountered as a top lung system pathogen in humans [6] and also a prevalent source of otitis media (OM) in newborns and children, contributing to 15–20% of severe OM episodes [7]. Moreover, *M. catarrhalis*-induced OM is thought to be moderate in compared with pneumococcal illness, multiple potential pathogens have already been discovered and it has been demonstrated that some epidermal constituents of *M. catarrhalis* produce inflammatory responses [8]. This bacterium can easily attach to the epithelium of a variety of nasal surfaces, including the lungs as well as the nasopharynx and elicits a powerful chronic inflammation defined by incursions of macrophages, lymphocytes and neutrophils into diseased tissue following infection, that is thought to be the etiology of OM and COPD relapses [9]. OM is common and widespread in developing nations and is a prominent reason of illness and death in children below the age of five [10]. It can cause major abnormalities in children’s linguistic, intellectual, academic and psychosocial development [11]. Additionally, *M. cattarhalis* is currently a good cause of around 10% of acute inflammatory comorbid conditions in individuals, the chronic obstructive pulmonary disease (COPD) [9]. In the United States, this bacterium is expected to trigger 2–4 million cases of chronic degenerative pulmonary disease in people annually [7].

In Bangladesh, several studies found substantial pervasiveness of OM in rural, urban and elementary school youngsters, with frequency rates of 43.2/1000, 32.6/1000 and 16.3/1000, correspondingly [11]. The detection of *M. catarrhalis* by several fatalities receptors (TLRs) including TLR4 and TLR9 induces the generation of erythrogenic mediators IL-6 and TNF- by the host defenses, according to several research studies [12, 13]. Also severe and recurring AOM infections have long been believed to include bacterial survival inside a biofilm due to their extremely resistant feature [14, 15]. Direct detection of bacterial biofilms in inpatient clinical specimens and the chinchilla model system of OM provide medical confirmation of bacterial biofilms [16, 17]. Several processes, along with epigenetic modification heterogeneity and slower spread of microorganisms inside this biofilm, postponed antibiotic infiltration through composite content, and the existence of viable cells [18] can dramatically increase antibiotic susceptibility in microbes within a biofilm congregation [19].

Moreover, some other preliminary research stated that *M. catarrhalis* exhibits a variety of antibiotic susceptibility strategies, including membrane porosity, active efflux mechanisms and alterations in antibacterial sites [20]. This bacteria shows that these strategies of susceptibility to beta-lactam antibiotics are usually related to type A serine β-lactamase enzyme [20, 21]. Beta-lactamases are enzymes generated by the preponderance of strains isolated from *M. catarrhalis* that allow them to withstand beta-lactam medications such as penicillin, amoxicillin and cephalosporins [22, 23]. In addition to this, the exterior layer porin M35 of *M. catarrhalis* is the factor that determines whether or not the bacteria are susceptible to aminopenicillins [24]. The resistance frequency of *M. catarrhalis* identified in infants to Beta-lactam antibiotics has attained 99% in China as a result of therapeutic experimental usage of antibiotics [25]. Furthermore, according to reports, *M. catarrhalis* appeared extremely susceptible to macrolide antibiotics, erythromycin and rokitamycin [25, 26]. In addition, some investigations have demonstrated that *M. catarrhalis* is sensitive to the antibiotics cefaclor, clarithromycin, azithromycin, doxycycline, co-trimoxazole, cefuroxime, cefixime and ceftriaxone, as well as ofloxacin and ciprofloxacin [27]. The percentage of resistance of *M. catarrhalis* to tetracycline has attained 65.7%, representing a remarkable rise in resistance [28].

In our current study, we gathered entire genomic sequences of *M. catarrhalis* strains from the NCBI Genome resource and built a phylogenetic tree to better comprehend the biological and developmental connection among the *M. catarrhalis* strains. We additionally obtained antibiotic resistance genes (AMR) of *M. catarrhalis* strains from many databases, including Comprehensive Antibiotic Resistance Database (CARD), Pathway-systems Resource Investigation Center (PATRIC) and ResFinder, as well as built a gene interaction network to analyze the multidrug susceptibility systems by utilizing those AMR genes. The application of gene interaction-based network is to identify whether the influence of genomic outcomes on biological activities is becoming progressively pertinent [29]. On the contrary, researchers have increasingly been interested in gene interaction networking investigations, which are thought to be valuable in understanding multidrug susceptibility in infectious and exploitative microorganisms, as well as other biological disorders [30, 31]. Currently, one of the most promising approaches to research the roles of genes and proteins, as well as their related collaborators, is to use gene interactions. It aids in the discovery of relevant biological information about AMR processes, which in turn assists in the identification of critical candidate genes or proteins in the cycle, as well as the development of innovative medications to combat ailments triggered by AMR virulent strains [32, 33]. The molecular linkages and processes of the AMR genes have been explored in this work, which will be crucial in the development of innovative and effective medications for the disease’s therapy. Moreover, we performed protein–protein interactions (PPIs) among the antibiotic-resistant genes, cluster investigation, recognition of hub proteins and pathway assessment to unveil the complicated biological framework, as well as gene linkage with resistance mechanisms and drug class. We employed a combination of clustering and topological techniques to uncover the physiologically significant genes involved in drug susceptibility pathways. Thus, we were able to pinpoint the origin of the antimicrobial-resistant genes as well as these gene interactions that occurred among the *M. catarrhalis* strains. All of these things were carried out to establish an affiliation in gene expression patterns in *M. catarrhalis*. However, the genes identified as prospective pharmacological targets can be employed to create novel molecules with pharmaceutical uses to reduce *M. catarrhalis* outbreaks. We anticipate that our findings will improve the knowledge about the molecular underpinnings of multidrug resistance pathways in *M. catarrhalis* bacterial strains.

## Materials and methods

### Genome information of *M. Catarrhalis*

From the NCBI Genome database (https://www.ncbi.nlm.nih.gov/genome), we have analyzed 215 strains of *M. catarrhalis*, but for the further analysis, we chose only the complete genome sequences of 12 strains of *M. catarrhalis.* The genome database is a comprehensive resource of NCBI that includes genome sequences and assembly metadata as well as mapping enrichment data such as variants, and indicators, including epigenomics data [34]. During the selection of genome sequences, chromosomes, scaffolds and contigs were not evaluated; only complete genome sequences were considered. Those strains were available from 1980 to 2022. Furthermore, most of the strains were from human middle ear and sputum sample.

### Identification of AMR genes

After retrieving the complete genomes of *M. Catarrhalis*, we extracted AMR genes from those complete genomes using repositories including the CARD, PATRIC as well as ARDB and investigated them. Here, CARD or Comprehensive Antibiotic Resistance Database (https://card.mcmaster.ca/) is an elevated data source concerning the underlying mechanism of antibiotic resistance genes. It is a vetted platform that provides standard DNA and protein sequences, identification models and computational tools in a regulated ontology that is the Antibiotic Resistance Ontology, which is established by CARD’s biocuration group for program configuration [35, 36]. Another side, the PATRIC [https://www.patricbrc.org/] provides a collection of potential pathogens information kinds that have been combined from various data sources. PATRIC is a collaborative initiative between the Bioinformatics Resource Center and the National Institute of Allergy and Infectious Diseases [37]. ResFinder (https://cge.cbs.dtu.dk/services/ResFinder/) is a repository that indexes antibacterial resistant genes discovered in the whole genome of bacteria. This is accomplished through the usage of BLAST [38]. In the end, we compiled the resistance mechanisms and drug classes associated with these AMR genes. Then, we employed Venny 2.1 (https://bioinfogp.cnb.csic.es/tools/venny/) to gather only the unique AMR gene. Venny 2.1 is a tool for mapping and comparing gene lists that may be used interactively [39, 40]. Then, we used these unique AMR genes to build gene interaction networks and for other further analysis.

### Phylogenetic tree construction

Most biological research requires an understanding of evolutionary interconnections between species. A reliable phylogenetic tree is essential for presuming the provenance of novel genes, identifying biochemical transformation, comprehension of morphological feature progression as well as recreating psychographic trends in diverged species [41]. We previously noted that we obtained a total of 12 complete-genome sequences of *M. catarrhalis*. The reference sequence was from the CCRI-195ME strain of *M. catarrhalis*. We employed Mega v11 software (https://www.megasoftware.net/) to do the phylogenetic investigation to determine the developmental and evolutionary connection between the *M. catarrhalis* strains. MEGA (Molecular Evolutionary Genetics Analysis) is a computer-based program for statistically analyzing molecular development, determining evolutionary process length and building phylogenetic relationships. It is open concerning safety and offers GDPR (General Data Protection Regulation) insurance to people all around the world [42]. However, the evolutionary history was estimated using the Neighbor-Joining statistical approach using 1000 bootstraps and then exported into iTOL (v. 6) (https://itol.embl.de/) for improved display. We also provided the length of each branch from the root. Here, the nodes in the phylogenetic tree indicate isolated strains, and the edges indicate the hamming distance between two strains.

### P‌PI network construction and visualization

PPIs regulate a vast variety of biological activities, and physiological activities notably tissue connectivity as well as developmental management [43]. To build the PPI network and identify the associated genes or protein databases, we utilized a well-known search program STRING (http://string-db.org). The STRING database plays an important role in assembling, evaluating and disseminating PPI data in a user-friendly and extensive way [44]. As a starting point, we provided STRING with a list of unique AMR genes so that it could look for their neighboring interactors. The extracted PPI network was generated with medium confidence (>0.40) in STRING. Finally, we employed Cytoscape_v3.9.1 to create a visual representation of the target network. Cytoscape (https://cytoscape.org/) is a prominent bioinformatics program for visualizing biological interactions and integrating data.

### Cluster formation and hub proteins extraction

Cluster analysis is a comprehensive method for combining expression profiles with protein–protein-interacting networks. In systems biology, it has a vital role in identifying regulatory components and estimating protein expression. We utilized the MCODE (https://baderlab.org/Software/MCODE) plug-in in Cytoscape to form the clusters. The MCODE plugin is intended to find densely connected zone also known as clusters in a biological network. In our current study, cluster formation was carried out applying the default parameter including degree score cutoff of 2, node score cutoff of 0.2 and K-Core of 2, and the maximum depth of 100 in the MCODE to verify the efficacy of interactive collaborators in the context of AMR gene expression. On the other hand, hub proteins, also known as key proteins, are characterized as proteins that have a significant degree of association on a wide range throughout the PPI network. In our ongoing study, we utilized the Cytoscape plug-in cytohubba (http://apps.cytoscape.org/apps/cytohubba) to find highly interconnected protein nodes as well as to investigate the network topology. The cytoHubba plugin is employed to obtain the protein nodes that are largely attributable inside the PPI network. Eleven topology analytical techniques are accessible in cytoHubba [45]. Our study included six analytical techniques from the Cytohubba plugin, including three locally ranked methodologies: degree, maximum neighborhood component (MNC) and maximum clique centrality (MCC), as well as three globally ranked methodologies: closeness centrality, betweenness and also the stress method. In the following step, the collected genes from the cytoHubba were submitted to jvenn (an interactive Venn diagram analyzer) **(**http://jvenn.toulouse.inra.fr/) for more analysis and the genes that were intersected among the six approaches of cytohubba were designated as significant hub proteins.

### Assessment of gene enrichment

Gene enrichment is a process of analyzing collections of genes using the gene ontology categorization system, whereby genes are classified into preset groups based on their operational features. Gene Ontology is categorized into three distinct activities: biological activities, cellular activities and molecular activities. Here, the term biological activities refer to the major cellular or metabolic significance of genes in coordination with other genes, cellular activities refer to the role of gene products within the cell, whereas molecular activities refer to the specific molecular function (MF) of a gene [46]. On the other hand, Kyoto Encyclopedia of Genes and Genome (KEGG) is also a biological explanatory scientific route database. KEGG pathway analysis aids in the discovery of linkages between core activities of critical genes, as well as in gaining a thorough understanding of the fundamental activities of genes [26, 47]. In this work, we retrieved GO keywords and KEGG pathway data from the STRING database and then utilized SRplot—Science and Research online plot (http://www.bioinformatics.com.cn/en) to display and further analyze them.

### Genes correlation with antibiotic resistance mechanism and drug class

Microbes develop methods to defend themselves against antimicrobial compounds, which are known as AMR mechanisms. These mechanisms have developed in bacteria due to a variety of reasons. A few of them include modifications of the permeability in the bacterial cell that constrain bacterial direct exposure to target areas, alternations of the enzyme’s catalytic activity, oversaturation of the intended enzymes, antimicrobial drugs modification and deterioration, development of metabolic processes other than those blocked by the medication, active efflux pump and so on [48]. On the other hand, penicillin and beta-lactam were the first antibacterial compounds identified [49]. These antibiotics were successful in treating bacterial infectious diseases. Other antibiotics, such as macrolides, aminoglycosides, chloramphenicol, tetracycline and streptothricin, as well as sulfonamide and trimethoprim, perform a significant function in the diagnosis and therapies of microbial pathogens [49]. These agents can repress the antimicrobial protein production while also interfering with DNA and RNA production, negatively affect the microbial cell wall production and prevent microbial cell energy biosynthesis [50]. In our ongoing study, during the process of collecting AMR genes from the CARD and PATRIC databases, we also put together a list of the resistance mechanisms and drug classes. Afterward, we reorganized all of the information, which included distinct genes, drug classes as well as resistance mechanisms, to execute the sunbursts plot using python. Python is a programming language that is employed to develop computer programs. Additionally, it is frequently utilized to perform automated operations, as well as statistical exploration [51]. However, this analysis helps to dig out the causes behind susceptibility, the enhanced strategy of detecting resistance when it emerges, alternative therapeutic choices for diseases triggered by resistant organisms, as well as attempts to mitigate and regulate the formation [52, 53].

## Results

### Collection of AMR gene

**Figure 2:**
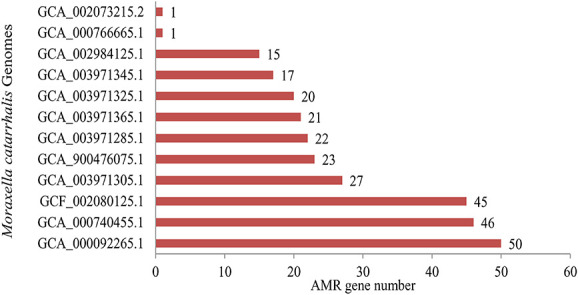
The number of AMR genes found in *M. catarrhalis*. The *X*-axis indicates the number of AMR genes, while the *Y*-axis indicates the name of the *M. catarrhalis* genomes.

We retrieved a total of 288 AMR genes from these 12 strains of bacteria (Figure 2). Among these AMR genes, 18 were from the card, 264 were from PATRIC and 6 were from ResFinder. Out of 288 collected resistance genes, 74 entries were found to be unique (Table S1 available online at http://bib.oxfordjournals.org/). However, these unique AMR genes were implemented to conduct additional exploration of this current study. The detailed information of bacterial genome size, genome coverage and gene number was provided in Table 1.

**Table 1:** Details annotation information of bacterial (*M. catarrhalis*) genome

Sl. No	Accession No.	Strain Name of *M. catarrhalis*	Genome Coverage	Genome size (Mb)	Host/source	Genes number	Ref.
1	GCF_002080125.1	CCRI-195ME (Reference)	Not provided	1.9	*Homo sapiens*/middle ear	1901	NCBI:txid480
2	GCA_000740455.1	25 240	317×	1.9	Unknown	1777	NCBI:txid480
3	GCA_000766665.1	25 239	211×	1.8	*H. sapiens*	1752	NCBI:txid480
4	GCA_002073215.2	FDAARGOS_213	1000.94×	1.9	*H. sapiens*	1747	NCBI:txid480
5	GCA_002984125.1	FDAARGOS_304	825.126×	1.9	*H. sapiens*/nose of a healthy pediatric carrier	1788	NCBI:txid480
6	GCA_003971285.1	74P50B1	30×	1.8	*H. sapiens*/sputum	1681	NCBI:txid480
7	GCA_003971305.1	142P87B1	26×	1.9	*H. sapiens*/sputum	1769	NCBI:txid480
8	GCA_003971325.1	46P58B1	24×	2.05	*H. sapiens*/sputum	1933	NCBI:txid480
9	GCA_003971345.1	74P58B1	31×	1.8	*H. sapiens*/sputum	1681	NCBI:txid480
10	GCA_003971365.1	5P47B2	29×	1.9	*H. sapiens*/sputum	1771	NCBI:txid480
11	GCA_900476075.1	NCTC11020	100×	1.9	Unknown	1748	NCBI:txid480
12	GCA_000092265.1	BBH18	Not provided	1.863	Unknown	1722	NCBI:txid1236608

### Phylogenetic tree analysis

**Figure 3:**
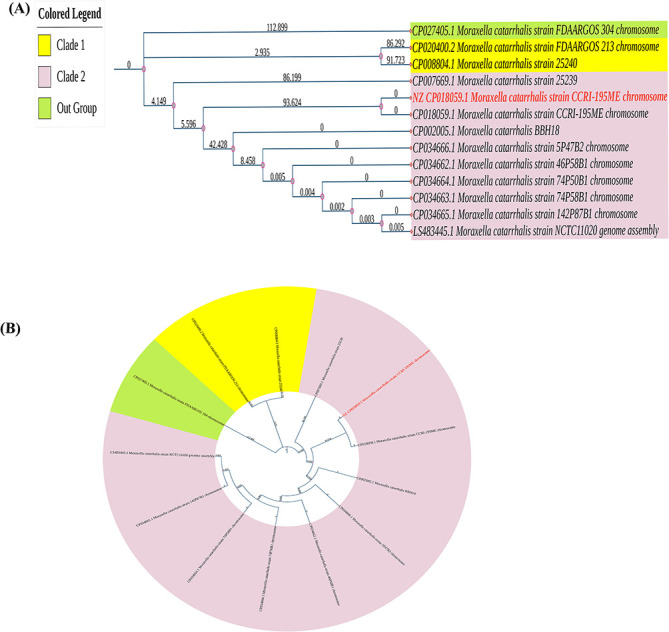
Phylogenetic tree (12 strains of *M. catarrhalis*)*.* (**A**) Rooted view and (**B**) Circular view. The spreading arrangement of two views of phylogenetic tree indicates how bacterial strains emerged from a prevalent origin. This phylogenetic tree was built using the genomes of 12 different *M. catarrhalis* strains using the Neighbor-Joining method and 1000 bootstraps. The strains are divided into two separate clades by the tree network (Clade 1 and Clade 2). In both views, the reference sequence *M. catarrhalis*, strain CCRI-195ME is marked (red). The edges of the phylogenetic tree indicate the hamming distance between two strains, and each node indicates a single strain.

We have indicated that there were 215 strains of *M. catarrhalis* in the NCBI up to 5 October 2022, but we analyzed only 12 complete strains with genome coverage of ≥20×, and size of the analyzed genomes varied from 1.8 to 2.05 Mbp (Table 1). We constructed a phylogenetic tree relying on the 12 complete genomes of *M. catarrhalis* and revealed the ancestral connection among them. Figure 3A and B represents the rooted and circular view of the phylogenetic tree, respectively. Among 12 strains, the phylogenetic tree revealed two major clades and one outgroup. Clade 1 consists of two strains and clade 2 consists of 9 strains of *M. catarrhalis*. Moreover, the out group strain was *M. catarrhalis* FDAARGOS_304 which was less linked to other strains. The reference sequence of *M. catarrhalis*, strain CCRI-195ME, has been highlighted in red in both images.

### P‌PI network analysis

**Figure 4:**
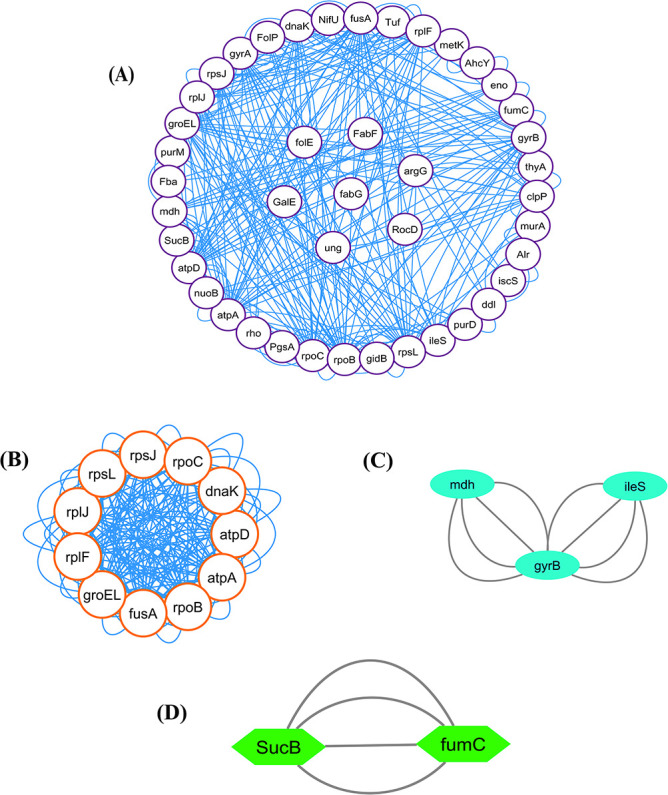
(**A**) PPI network. In this network, AMR genes are indicated as white nodes and gene connections are indicated by blue edges. Furthermore, cluster analysis was performed to produce relevant and persistent sets of comparable genes for biological identification and evaluation. (**B**) Cluster C1 (11 nodes, 192 edges), (**C**) Cluster C2 (3 nodes, 8 edges) and (**D**) Cluster C3 (2 nodes, 4 edges) show the highly connected proteins among the PPI network.

In our present study, STRING was used to generate the PPI network through the unique genes. In a PPI network, proteins or genes are denoted as nodes, while interconnections between these nodes are denoted as edges. The PPI network that we extracted in our current analysis contains 43 nodes and 288 edges. Moreover, the clustering coefficient of the network was 0.414. Figure 4A depicts the PPI network. It represents the connectivity of *folE, FabF, argG, RocD, ung, fabG* and *GalE* genes to other nodes within the network.

### Cluster analysis and hub proteins identification

**Figure 5:**
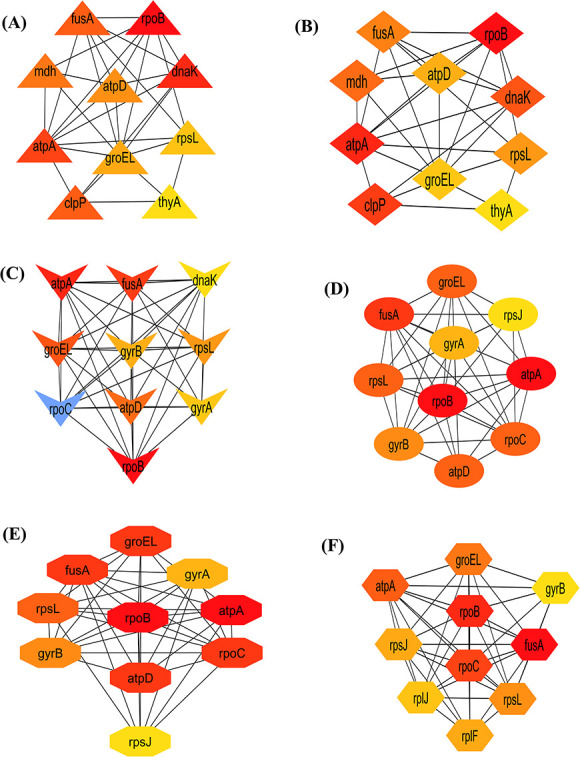
Hub gene identification using six Cytohubba plugins in Cytoscape. (**A**) Stress, (**B**) Betweenness, (**C**) Closeness, (**D**) Degree, (**E**) MNC and (**F**) MCC. These hub genes are referred to as highly linked fundamental nodes in a large-scale-free PPI network that includes diverse functional partners that combine various network components. Color gradients indicate the higher to lower value from red to yellow.

**Figure 6:**
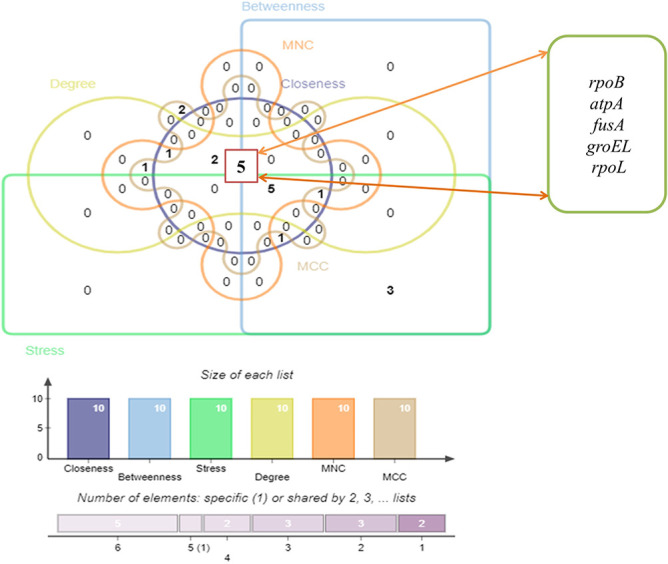
Interpretation of JVENN analysis. The overlapped region comprises five genes *(rpoB, atpA, fusA, groEL* and *rpoL)* common among hub genes gathered using six Cytohubba approaches (Stress, Betweenness, Closeness, Degree, MNC and MCC).

We detected three significant clusters in the PPI network employing the MCODE plugin of Cytoscape. Within the clusters, the 1st cluster (C1) comprised 11 nodes and 192 edges (score: 25.517), the 2nd cluster (C2) comprised 3 nodes and 8 edges (score 2) as well as the 3rd cluster (C3) comprised 2 nodes and 4 edges (score 2). The three clusters are shown in Figure 4B–D, respectively. In addition, Table 2 displays the genes involved within those three clusters. Furthermore, we discovered hub proteins by employing six Cytohubba plugin methodologies, comprising Stress, Betweenness, Closeness, Degree, MNC and MCC (Figure 5). Then, Jvenn analysis was performed on six classes of hub proteins obtained using the aforementioned methods. From the Jvenn analysis, we noticed that five proteins were shared by all methods namely *rpoB, atpA, fusA, groEL* and *rpoL*. These five proteins were identified as significant hub proteins. The Jvenn diagram is shown in Figure 6, and Table 3 shows the topological properties of significant hub proteins. On the contrary, these hub proteins were also detected in cluster 1, cluster 2 and cluster 3, indicating that they were the most crucial hub proteins. In Table 2, we have highlighted the hub genes which were presented in those three clusters.

**Table 2:** Identification of gene clusters (C1–C3) of *M. catarrhalis* from the PPI network

Cluster	Score (density)	Nodes	Edges	Gene name
C1	25.517	11	192	***rpsJ, rpoC, dnaK, atpD, atpA, atpA, fusA, groEL, rplF, rplJ, rpsL.***
C2	2	3	8	***mdh, gyrB**, ileS.*
C3	2	2	4	*SucB, fumC.*

Note: Bold symbol indicates the potential hub genes.

**Table 3:** Topological characteristics of unique hub genes

Gene Name	MCODE Cluster	Stress	Betweenness	Closeness	Degree	MNC	MCC
*rpoB*	NIC	8824	249.4637201	28.58333333	38	19	49 842
*dnaK*	C1	8640	181.5186545	24.16666667	22	10	5769
*atpA*	C1	5896	197.1844322	28.33333333	38	19	47 752
*clpP*	NIC	5584	188.6512432	22	14	5	12
*fusA*	C1	5584	138.2553313	26.91666667	32	15	50 427
*mdh*	C2	5320	153.4120443	23.3333	20	9	173
*atpD*	C1	4904	115.8893319	26.08333333	30	15	6676
*groEL*	C1	4880	106.0333377	26.66666667	30	15	47 654
*rpsL*	C1	4512	122.0596986	25.66666667	30	14	43 939
*thyA*	NIC	4200	103.3791209	20.5	12	4	10
*rpoC*	C1	3072	75.43951	25.91666667	30	15	49 706
*gyrB*	C2	3512	66.69975	25.41666667	26	13	8070
*gyrA*	NIC	2476	54.03081	24.58333333	24	12	2168
*rpsJ*	C1	400	8.7243	23.3333	22	11	41 784
*rplF*	C1	224	3.08944	23	20	10	41 784
*rplJ*	C1	184	3.00269	22.83333	20	10	41 760

### Functional enrichment analysis

**Figure 7:**
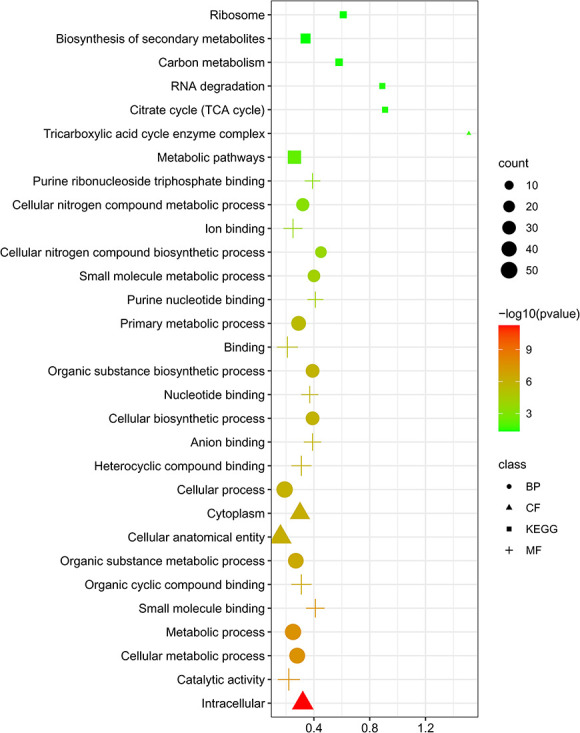
Functional Enrichment analysis (Gene Ontology and KEGG pathway) of AMR genes. The top 24 GO keywords and top 6 KEGG functional pathways are shown by the study of AMR genes. In the bubble plot, circular-shaped indicates BP; triangle-shaped represents cellular component (CP); plus sign indicates MF and square-shaped indicates KEEG pathway.

Functional enrichment was performed by employing unique AMR genes. In this present study, GO and KEGG pathway findings revealed some biological, cellular and molecular processes as well as some multidrug susceptible mechanisms that were strongly linked with AMR genes of *M. catarrhalis*. In this case, the outputs of GO enrichment provided a biological process (BP) that was significantly correlated with cellular metabolic process, cellular biosynthetic process, organic substance metabolic process, primary metabolic process, Cellular nitrogen compound biosynthetic, etc*.* MFs enriched with catalytic activity, organic cyclic compound binding, heterocyclic compound binding, anion binding and purine ribonucleoside triphosphate binding. Furthermore, intracellular, cytoplasm, cellular anatomical entity and tricarboxylic acid cycle enzyme complex principally involved with the cellular component. Meanwhile, the KEGG pathway enrichment study discovered that metabolic pathways, citrate cycle (TCA cycle), RNA degradation, carbon metabolism, biosynthesis of secondary metabolites, etc*.*, were substantially related to the AMR genes (Figure 7). Furthermore, Tables 4 and 5 and Table S2, available online at http://bib.oxfordjournals.org/, provided the tabular representation of the GO and KEGG pathway.

**Table 4:** Most significant relevant pathway of each GO term of AMR genes

Category	GO ID	GO Terms
BP	GO:0008152	Metabolic process
	GO:0044237	Cellular metabolic process
	GO:0071704	Organic substance metabolic process
	GO:0009987	Cellular process
	GO:0044249	Cellular biosynthetic process
	GO:1901576	Organic substance biosynthetic process
	GO:0044238	Primary metabolic process
	GO:0044281	Small molecule metabolic process
	GO:0044271	Cellular nitrogen compound biosynthetic process
	GO:0034641	Cellular nitrogen compound metabolic process
Molecular function	GO:0036094	Small molecule binding
	GO:0003824	Catalytic activity
	GO:0097159	Organic cyclic compound binding
	GO:1901363	Heterocyclic compound binding
	GO:0043168	Anion binding
	GO:0000166	Nucleotide binding
	GO:0005488	Binding
	GO:0017076	Purine nucleotide binding
	GO:0043167	Ion binding
	GO:0035639	Purine ribonucleoside triphosphate binding
Cellular function	GO:0005622	Intracellular
	GO:0005737	Cytoplasm
	GO:0110165	Cellular anatomical entity
	GO:0045239	Tricarboxylic acid cycle enzyme complex

**Table 5:** Most significant relevant pathway KEGG pathway analysis along with term ID and description

Pathway	Term ID	Term description
KEGG	mct01100	Metabolic pathways
	mct00020	Citrate cycle (TCA cycle)
	mct03018	RNA degradation
	mct01200	Carbon metabolism
	mct01110	Biosynthesis of secondary metabolites
	mct03010	Ribosome

### Genes correlation with resistance mechanisms and drug class analysis

**Figure 8:**
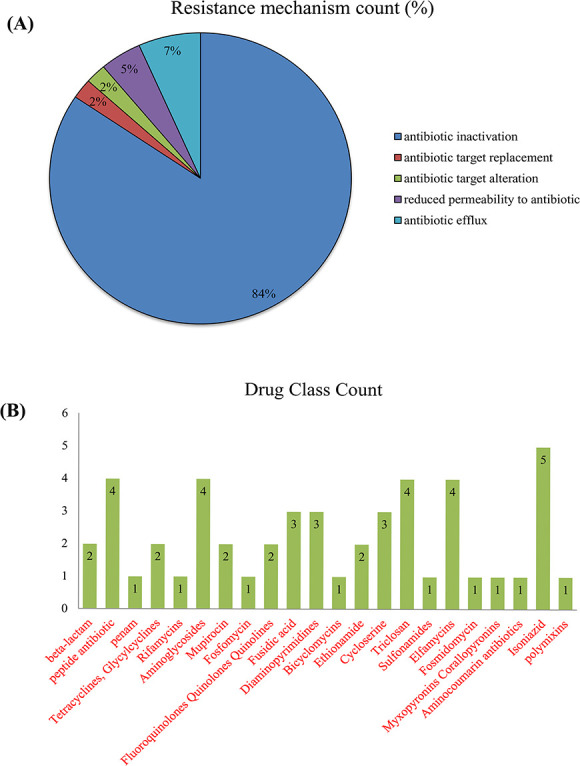
*Moraxella catarrhalis* significantly utilize several kinds of AMR mechanisms and drug classes. (**A**) Resistance mechanisms count and (**B**) Drug Class count.

**Figure 9:**
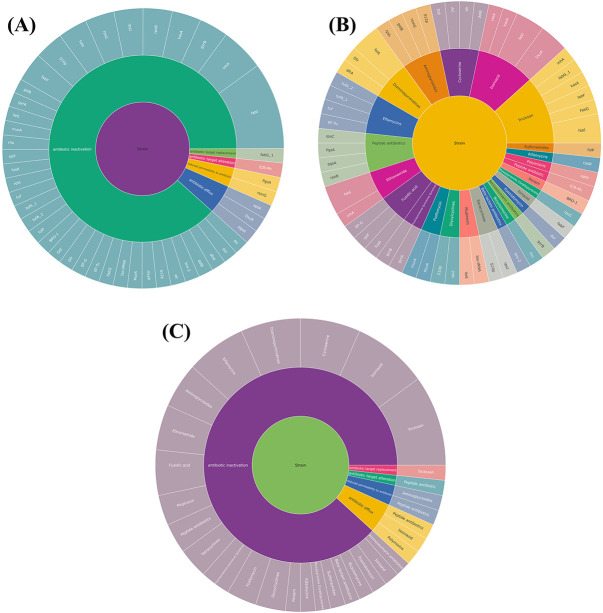
Sunburst plots were used to build interactions between different gene classes, drug classes and resistance mechanisms in networks. **(A)** The relationship between resistance mechanisms corresponding to their genes. (**B**) The relationship between drug classes corresponding to their genes. (**C**) The relationship between resistance mechanism and drug class.

We used a sunburst plot to identify the genes associated with resistance mechanisms and drug classes in *M. catarrhalis*. According to the plot, various *M. catarrhalis* strains contain many genes and employ various mechanisms and techniques to increase their resistance capacities against various multiple medications. Antibiotic inactivation, antibiotic target replacement and alteration, reduced permeability to antibiotics and antibiotic efflux pump are some of the resistance mechanisms employed by *M. catarrhalis*. Figure 8A and B indicates the different types of resistance mechanisms and drug classes exploited by *M. catarrhalis*, as well as their numbers. The fabG-1 gene is mainly linked to fatty acid biosynthesis, not target replacement, while ICR-Mc lacks sufficient characterization, and its role in antibiotic target alteration remains unconfirmed without experimental evidence. Furthermore, we observed that *M. catarrhalis* has resistance activity in a variety of antibiotics, including beta-lactam, peptide, penam, tetracyclines, rifamycins, aminoglycosides, fosfomycin and others, according to the CARD and PATRIC databases (Table S3 available online at http://bib.oxfordjournals.org/). Figure 9A–C represents the whole relationship between the genes with resistance mechanisms and drug classes.

## Discussion

AMR is the inability of microbes to adapt to various antibiotics, which makes it more difficult to prevent infectious diseases and also raises the overall probability of disease transmission, serious morbidity and fatality. Several AMR genes perform a vital function in developing this resilience to several anti-disease medications in bacterial pathogens. Currently, it has become a persistent danger to our capacity to cure prevalent diseases due to the formation and transmission of drug-resistant bacteria. In the current work, we have predicted some AMR genes in the pathogenic bacterial strain M. catarrhalic via computational approaches. *Moraxella catarrhalis* is typically detected in humans as a symbiotic and mucosal parasite that might cause OM, chronic obstructive pulmonary disease (COPD), ocular infection, sinusitis as well as infrequently laryngitis in humans. As previously mentioned, we gathered 288 AMR genes from the strains of *M. catarrhalis via* several databases; then, we conducted phylogenetic analysis, protein interaction network analysis, clustering analysis, hub protein identification and pathway analysis of AMR genes, to predict drug that are confer to resistance. In addition to this, we hypothesized an approach to identify how AMR genes are linked to diverse antibiotic resistance strategies as well as different categories of antibiotics.

Environmental microorganisms, including other species on the earth, are vulnerable to the processes of molecular diversity. Genome sequencing provides information on the molecular diversity of organisms through the analysis of those sequences. More than that, significant perspectives on microbiological as well as pragmatic applications such as disease identification might be gained by integrating the DNA sequencing of microorganisms and developmental modeling with phylogenetic studies [54]. The phylogenetic analysis of the data from our current investigation indicated the link between the *M. catarrhalis* strains. The strain CCRI-195ME of *M. catarrhalis*, which was considered a reference sequence, was highly correlated with other strains found in GenBank such as *M. catarrhalis* FDAARGOS 304, *M. catarrhalis* NCTC11020, *M. catarrhalis* 142P87B1, *M. catarrhalis* 74P58B1, *M. catarrhalis* 46P58B1 and *M. catarrhalis* BBH18. Among these strains, *M. catarrhalis* CCRI-195ME was the first fully sequenced genome with the *modM3* allele, derived from the inner ear of a 16-month aged infant predisposed to OM [55]. About 80% of the total children have experienced a minimum of one episode of OM by the time they are 3 years old, resulting in the most frequent pediatric illnesses [56]. Additionally, this bacterium is currently regarded to be the 2nd highest prevalent reason for the deterioration in COPD [57]. Unfortunately, the specific function of bacteria is not well known and is a contentious topic. Although the importance of developing a vaccine that is effective against *M. catarrhalis*, none of the contenders have advanced to the medical testing stage. Because of this, we must interpret the diversity of *M. catarrhalis* to develop an improved insight of the epidemiological data as well as the propagation of genes involved in pathogenicity aspects, which will assist in the development of vaccines [57].

Furthermore, the PPI network that was analyzed in this study showed that the genes *folE, FabF, argG, RocD* and *fabG* are all connected to neighboring nodes in the network. Previous findings have suggested that the *folE* motif might act as a potent riboswitch for the structural intergenic RNAs found in microbial noncoding regions [58]. This *folE* motif, which is typically located on the proximal of *folE* genes, follows a straightforward construction [59]. Furthermore, these genes encode enzyme that catalyzes the initial stage of the *de novo* folate manufacturing process in bacteria [58]. *FabF* and *fabG* perform a critical part in the bacterial process of fatty acid production. For the discovery of novel antibacterial drugs, the fatty acid production route is substantially underutilized. Initially, *FabH* catalyzes the condensation process between acetyl CoA and malonyl-acetyl carrying molecule to create acetoacetyl-ACP, which is crucial for the commencement of fatty acid biosynthesis [60]. In this cycle, *FabF* generates -ketoacyl-ACP, which boosts the rate of fatty acid biosynthesis of different lengths for activation by the microorganism [61, 62]. But by producing butyryl-ACP, *fabG* reduces the rate of fatty acid biosynthesis [60]. Other side, *argG* gene, which encodes argininosuccinate synthetase enzyme, is required in arginine biosynthesis in bacteria [63]. Arginine performs a significant function in the metabolic process of *M. catarrhalis* [64]. In *Bacillus subtilis*, a gene named *rocD* was discovered adjacent the *rocR* gene which was also present in *M. catarrhalis* [65]. The *rocD* gene produces an enzyme that is structurally analogous to eukaryotic ornithine aminotransferases [66]. Therefore, these genes perform many crucial roles in the metabolic, cellular and BPs that take place in microorganisms. In our investigation, we predicted that GO pathways may be related to antibiotic-resistant processes including BPs, cellular components and molecular activities were significantly elevated. BPs were mostly related to the cellular metabolic process (GO:0044237), organic substance metabolic process (GO:0071704), cellular biosynthetic process (GO:0044249), organic substance cellular biosynthetic process (GO:0044249), organic substance biosynthetic process (GO:1901576), primary metabolic process (GO:0044238), small molecule metabolic process (GO:0044281), cellular nitrogen compound biosynthetic process (GO:0044271) and cellular nitrogen compound metabolic process (GO:0034641). GO term enrichment analysis revealed associations with general cellular processes such as metabolism and biosynthesis. While these terms are commonly enriched in bacterial genomic datasets, their presence does not necessarily indicate a direct mechanistic link to antimicrobial resistance. Additionally, disruptions in bacterial metabolic equilibrium have substantial side effects on therapy concerning drugs [67]. On the other hand, the primary metabolic process entails biochemical events and mechanisms which generate substances during regular anabolic and catabolic events, and the organic substance metabolic process entails the series of biochemical events that an organic matter, which can be thought of as like any molecule or other unit that contains carbon, is involved in [68, 69].

Remarkably, both the cellular nitrogen compound biosynthetic process and the cellular nitrogen compound metabolic process are implicated in the formation of nitrogen, a crucial component for the production of, amino acids, proteins, different types of enzymes, DNA and RNA in all microorganisms [70]. Among the cellular function terms cellular anatomical entity (GO:0110165), tricarboxylic acid cycle enzyme complex (GO:0045239), cytoplasm (GO:0005737), etc*.*, were mostly enriched. Similarly, the following MFs were found to be accumulated: small molecule binding (GO:0036094), catalytic activity (GO:0003824), organic cyclic compound binding (GO:1901363), nucleotide binding (GO:0000166), organic cyclic compound binding (GO:0097159), heterocyclic compound binding (GO:1901363) anion binding (GO:0043168), purine nucleotide binding (GO:0017076), purine ribonucleoside triphosphate binding (GO:0035639) and ion binding (GO:0043167). Currently, nanomaterial-based therapeutics are intriguing methods for combating complicated bacterial infestations, as they can circumvent established processes linked with accumulated antibiotic resistance [71]. Moreover, heterocyclic compounds, such as thiazole, benzothiazole and thiazolidinone, have been produced over the previous decades in an effort to acquire novel antibiotics capable of treating conditions triggered by antimicrobial resistant bacterial strains [72]. We also uncovered KEGG pathways associated with the citrate cycle (TCA cycle), RNA degradation, carbon metabolism and secondary metabolite production. In microorganisms, RNA can be degraded *via* multiple processes. Controlling gene expression relies heavily on RNA synthesis and degradation, which plays an important part in the molecular process [73]. Additionally, carbon metabolism is essential for bacterial proliferation [74]. Since bacteria cannot manufacture their food, they must rely on the source of carbon for the synthesis of energy and metabolic substances [75]. These substances are required for the production of anabolic subunits, which are then transformed into polymers, including organic molecules (proteins, nucleotides), as well as elements of the complicated cell membrane [75, 76]. Mainly the citrate cycle also referred to as the Krebs cycle, is the fundamental route through which cells obtain their supply of energy and is an essential component of cellular breathing. This cycle is also employed as the foundation for secondary metabolite synthesis because its byproducts are utilized as substrates in the production of metabolites; numerous molecular systems are linked to the CAC, forming a biochemical circuit [77].

Aforementioned, we identified three important clusters, which are referred to as C1, C2 and C3, and also detected hub proteins using six Cytohubba plugin approaches. In addition, we detected several hub genes that were unique among six approaches including *rpoB, dnaK, atpA, clpP, fusA, mdh, atpD, groEL, rpsL, thyA, rpoC, gyrB, gyrA, rpsJ, rplF* and *rplJ.* Among these genes, *dnaK, atpA, fusA, atpD, groEL, rpsL, rpoC, rpsJ, rplF* and *rplJ* were present in C1 as well as *mdh and gyrB* were present in C2. We predicted that these hub genes may be significantly correlated with various types of drug resistance mechanisms along with drug classes. These mechanisms are carried on by changes to the drug, changes to the antimicrobial targets, restricted access to the target, or even a mixture of these processes [78–80]. Because of its capacity to manufacture BRO-lactamase, *M. catarrhalis* is reported to be resistant to penicillin as well as the foundation follows cephalosporins; nevertheless, it is normally sensitive to additional drugs, notably fluoroquinolones [81, 82]. However, in our analysis, we predicted some potential resistance mechanisms including drug target alteration, antibiotic inactivation, antibiotic target replacement and reduced permeability to antibiotics. Antibiotic inactivation was carried out by 36 genes (fabI, inhA, gyrB, kasA, rpoB, rpsJ, rpoC, folA, S10p, fabF, gidB, gyrA, ileS, murA, rho, rplF, fusA, rpsL, tuf, tufA_1, tufA_2, folP, BRO-1, Ddl, Dfr, EF-G, EF-Tu, fabG, IsotRNA, OxyR, S12p, alr, bro-2, ddlB, dfrA and dxr) which were predicted in our present study. The fabG-1 gene is primarily involved in fatty acid biosynthesis and is not directly implicated in target replacement. Similarly, ICR-Mc remains insufficiently characterized in current literature, and its role in antibiotic target alteration cannot be confirmed without experimental validation.

PgsA is involved in membrane phospholipid biosynthesis and may indirectly affect antibiotic permeability under certain conditions, although its direct role in resistance to peptide or aminoglycoside antibiotics remains unconfirmed. On the other hand, rsmG (gidB) encodes a 16S rRNA methyltransferase; mutations in this gene have been associated with low-level resistance to aminoglycosides via target modification rather than reduced permeability. The fabG-1 gene is primarily involved in fatty acid biosynthesis and is not directly implicated in target replacement. Similarly, ICR-Mc remains insufficiently characterized in current literature, and its role in antibiotic target alteration cannot be confirmed without experimental validation as was previously stated.

In addition. the PgsA, OxyR and eptA hub genes are not involved in efflux mechanisms. The eptA gene modifies lipid A and is associated with colistin resistance in some Gram-negative bacteria. On the other hand, PgsA is involved in phospholipid biosynthesis while OxyR is an oxidative stress response regulator. Various studies concluded that the presence of efflux pumps, which potentially provide resistance to macrolides, b-lactams, macrolides, tetracyclines, aminoglycosides and fluoroquinolones, is common in MDR microorganisms [83, 84].

To prevent the propagation of the infection, prompt detection with appropriate management is crucial. Singpanomchai *et al.* [85] and Guitor [86] have indicated in their studies that infections that are prominent in respiratory regions cause significant *rpoB* gene alterations in RNA polymerase during RNA production, leading to alter drug binding site and further resistance to rifamycin. Mujawar *et al.* [87] revealed that *DnaK* is essential for antibiotic resistance through a variety of assessments using microbial extracts. The nucleotide alterations from cytosine to adenine on *DnaK* gene were identified by the SNPs discovered in the individual samples that potentially caused protein denaturing [87]. In the same way, the *DnaK-GroEL* interaction may play a significant contribution to antibiotic resistance in pathogenic microorganisms [88]. The *GyrA* and *GyrB* genes encode DNA gyrase enzyme that contributes to the key methods of quinolone (QN) susceptibility and comprises a reduction in interaction propensity to QNs caused by amino acid change in the QRDR (quinolone resistance-determining region) [89]. On the other hand, through the evaluation of the gene’s genomic structure, many researchers demonstrated in their research that the *rpsL, ClpP**,** rpsJ* and *rplJ* function as potential antibiotic resistance genes [90–94].

In contrast, the major cause of antibiotic resistance may also be mutations. The probability in which identifiable mutations appear in a microbial species that exposed to a specific therapeutic dose shows how the evolution rate is usually characterized in the perspective of antimicrobial sensitivity. Numerous investigations have demonstrated that even an one amino acid change can result in the development of beta-lactam resistance in isolates that are extremely sensitive to penicillin have additional penicillin-binding proteins changes than bacteria that are intermediately resistant [95]. Furthermore, Jacobs and Micheael R showed in their study the activation of an erythromycin ribosomal methylation gene leads in transcription factors alteration of 23S ribosomal RNA, which prevents the macrolide from attaching to the ribosome [96]. They discovered that it is the source of the majority of pneumococcal macrolide susceptibility. However, many clinical studies have revealed that *M. catarrhalis* isolates are extremely sensitive to macrolides. Even several studies have found that a mutation in the *TonB*-dependent receptor expressing gene *MCR 0492* may be linked to macrolides susceptibility in *M. catarrhalis* strains [97]. Kasai *et al.* [98] used naturally occurring erythromycin-resistant variants to study the impact of alterations in the 23S rRNA gene and also the *L4* and *L22* ribosomal subunits. Additionally, the development of rising macrolide-susceptibility *M. catarrhalis* may be attributed to the *A2330T* Mutation in the 23S rRNA gene [99]. Therefore, we may conclude that alterations in 23S rRNA are the major cause of macrolide resistance. On the other hand, fluoroquinolone resistance is induced by significant mutations in the DNA gyrase enzymes, which can potentially be induced by the emergence of efflux pumps within the bacterium [100]. Warner *et al.* [101] attempted to demonstrate in their work that therapeutically significant mutations that induce derepression of the *Neisseria gonorrhoeae MtrC-MtrD-MtrE* Efflux pump system provide various rates of antimicrobial sensitivity. Likewise, mutation in *Mycobacterium tuberculosis* may be the cause of the individual’s inherent resistance to numerous antibiotics, which reduces the amount of drugs that are accessible for therapy [102]. Antibiotic resistance mutations may have cytoprotective impacts, resulting in a decrease bacterial viability, as measured, for example, by a decrease in laboratory multiplication efficiency.

Thus, our findings reveal several mechanisms of antibiotic resistance and associated interaction networks, predicted entirely through computational methods. But we have faced several limitations of this study: (i) Our identified several GO pathways reflect the multifunctional roles of some AMR-related genes and donot imply a specific mechanistic link between core metabolism and resistance; (ii) Mentioned hub genes such as rpoB, atpA, fusA, groEL and rpoL are essential housekeeping genes, encoding highly conserved components involved in core cellular processes; (iii) Their identification as hub genes in the PPI network reflects their centrality in cellular function, rather than their specific involvement in AMR; and (iv) Directly targeting such essential and conserved proteins carries a high risk of host toxicity and lacks pathogen specificity, making them suboptimal for drug development in their native form. We believe that these observations will offer clarity on the underlying process that leads to AMR in *M. catarrhalis.* In near future, we will be validated these antibiotic susceptibility patterns in wet lab experiments. Firstly, we will collect sample from patients or pure culture in hospitals or clinic. Secondly, we will analyze bacterial growth curve for the test of antibiorgram susceptibility patterns and then validate the gene expression patterns (potential hub gene) through Polymerase Chain Reaction analysis. Finally, we will sequence the potential bacteria based on 16sRNA analysis for the identification of possible new bacterial strains.

## Conclusion

Antibiotic resistance in virulent bacteria is a prominent cause for concern all around the world. It is a persistent issue in the medical sector. In this work, we investigated and predicted several AMR genes and pathways in the virulent strain of M. catarrhalis using genomics interaction and systems biology approaches. The AMR genes, in conjunction with their resistance mechanisms through the inactivation of antibiotics, target replacement of antibiotic, target alteration and reduced permeability to antibiotic. Various antibiotics, notably beta-lactam, tetracyclines, glycylcyclines, aminoglycosides and fosfomycin are sensitive to these mechanisms. In addition, the clustering approach uncovered gene sets that are intricately linked to one another. The genes *rpoB, atpA, fusA, groEL* and *rpoL* have quite a significant contact as well as can be more important for figuring out how the predicted hub genes from PPI network reflects their centrality in cellular function, rather than their specific involvement in antimicrobial resistance via only computationally analysis. Therefore, we believe that the findings that we have provided in this study will provide researchers with a solid foundation upon which to build their investigations into therapeutic approaches for the management of *M. catarrhalis* epidemics via a series of phenotypic assays including MIC testing, and gene expression analysis (RT-qPCR). We have analyzed only complete annotation of Genbank files datasets. In addition, we have searched three antibiotic resistance database, while several databases are available. There is also a lack of systematic antibiotic resistance gene collection predicted by different databases and further evaluate the reliability of wet lab experiment.

**Key Points**

- The fundamental underpinnings of antibiotic susceptibility in the pathogenic bacterial strain *Moraxella catarrhalis* were discovered using genomic interaction study relying on system biology.
- The PPI network uncovered important hub-proteins that reflects their centrality in biological function, rather than their direct link between bacterium and AMR systems.
- Cluster analysis was used to generate useful and long-lasting groupings of similar factors for biological characterization and assessment that coincided with resistance mechanisms along with corresponding drug classes.
- Functional enrichment was carried out to identify several biological processes, cellular functions and molecular processes that reflect only multifunctional roles of some AMR-related genes without direct mechanisms of resistance.
- The molecular connections underlying mechanisms of the AMR genes were investigated in this study, which is important in the creation of new and efficient treatments for infectious diseases.

## Supplementary Material

Supplementary_File_elaf027

## Data Availability

The used datasets are publicly available and the research data can be accessible on request.

## Notes

**Sadia Afrin Bristy** currently works in Bioinformatics and Biomedical Research Network of Bangladesh, Dhaka, Bangladesh. Her research interests include computational biology, system biology, drug design and bioinformatics.

**Md. Arju Hossain** is a thesis student (Master of Science) of Biotechnology and Genetic Engineering, Mawlana Bhashani Science and Technology University. He focuses on his study in numerous areas, including System Biology, Bioinformatics, Pharmacogenomics, Immunology, Metabolomics and Microbiology.

**Dr. Md Habibur Rahman** has received his B.Sc in Computer Science and Engineering (CSE) from the Dept. of Computer Science and Engineering, Islamic University, Kushtia-7003, Bangladesh in 2006. Later on, he has finished his M.Sc. in CSE from the same University in 2007. Recently, he has received his PhD in Pattern Recognition and Intelligent Systems from The University of Chinese Academy of Sciences, Beijing, China in 2020. His research interest encompasses Bioinformatics, Computational Biology, Machine Learning, Deep Learning and Drug Discovery. He is currently working as an Associate Professor at the Dept. of CSE, Islamic University, Kushtia-7003, Bangladesh. He is also investigating some projects as principal investigator and co-investigator and supervising several undergraduate and post-graduate students. He has presented several papers in peer-reviewed journals and has published numerous studies in science cited journals. He was an Awardee of the CAS-TWAS Presidential Fellowship, from 2016 to 2020.

**Md Imran Hasan** is a graduate student of the Department of Computer Science and Engineering, Islamic University. His research priorities include Bioinformatics, Machine learning, Deep learning, Medical Imaging, and Networking Dr. S. M. Hasan Mahmud received a Ph.D. degree in Computer Science and Technology from the Universityof Electronic Science and Technology of China, China. He received his B.Sc.degree in software engineering from the Shenyang University of Chemical Technology, China, and his M.Sc. degree in software engineering from Hohai University, China. He is working as an Assistant professor with the Department of Computer Science, American International University Bangladesh (AIUB). Besides he is also a research scientist at Mahidol University, Thailand. Before joining AIUB, he served as a Lecturer in the Software Engineering department at Daffodil International University, Bangladesh. He has published many research articles in reputed journals and conferences. His research interests include machine learning, deep learning, bioinformatics, drug discovery, and pattern recognition. He received several Best Paper Awards from the IEEE Conferences.

**Dr Mohammad Ali Moni** holds a PhD in Artificial Intelligence & Clinical Informatics in 2015 from the University of Cambridge, UK followed by postdoctoral training at the University of New South Wales, University of Sydney Vice-chancellor fellowship, and Senior Data Scientist at the University of Oxford. Dr Moni then joined as a research senior lecturer University of Queensland in 2021. He is an Artificial Intelligence, Computer Vision & Machine learning, Digital Health Data Science, Health Informatics and Bioinformatics researcher developing interpretable and clinical applicable machine learning and deep learning models to increase the performance and transparency of AI-based automated decision making systems. He has published over 200 journal articles in many top tier journals.
