# Supplementary material for: An integrated complete-genome sequencing and systems biology approach to predict antimicrobial resistance genes in the virulent bacterial strains of Moraxella catarrhalis
Source: Brief Funct Genomics. 2026 Feb 2;25:elaf027. doi: 10.1093/bfgp/elaf027 (PMC12930093; doi:10.1093/bfgp/elaf027)
Supplement: Supplementary_File_elaf027 [file Supplementary_File_elaf027.docx]

**Table S1: Details information of data collection**

| **Genbank ID** | **Strain** | **Size (M bp)** | **Resistance gene name** | **AMR gene family** | **Drug class** | **Resistance mechanism** |
| --- | --- | --- | --- | --- | --- | --- |
| **Card Database** | | | | | | |
| ASM9226v1 | BBH18 | 1.863 | ICR-Mc | intrinsic colistin resistant phosphoethanolamine transferase | peptide antibiotic | antibiotic target alteration |
| ASM74045v1 | 25240 | 1.942 | ICR-Mc | intrinsic colistin resistant phosphoethanolamine transferase | peptide antibiotic | antibiotic target alteration |
| ASM76666v1 | 25239 | 1.88 | ICR-Mc | intrinsic colistin resistant phosphoethanolamine transferase | peptide antibiotic | antibiotic target alteration |
| ASM207321v2 | FDAARGOS_213 | 1.908 | ICR-Mc | intrinsic colistin resistant phosphoethanolamine transferase | peptide antibiotic | antibiotic target alteration |
| ASM208012v1 | CCRI-195ME (Reference) | 1.994 | ICR-Mc | intrinsic colistin resistant phosphoethanolamine transferase | peptide antibiotic | antibiotic target alteration |
| ASM298412v1 | FDAARGOS_304 | 1.942 | ICR-Mc | intrinsic colistin resistant phosphoethanolamine transferase | peptide antibiotic | antibiotic target alteration |
|  |  |  | BRO-1 | BRO Beta-lactamase | penam | antibiotic inactivation |
| ASM397128v1 | 74P50B1 | 1.832 | ICR-Mc | intrinsic colistin resistant phosphoethanolamine transferase | peptide antibiotic | antibiotic target alteration |
|  |  |  | BRO-1 | BRO Beta-lactamase | penam | antibiotic inactivation |
| ASM397130v1 | 142P87B1 | 1.909 | ICR-Mc | intrinsic colistin resistant phosphoethanolamine transferase | peptide antibiotic | antibiotic target alteration |
|  |  |  | BRO-1 | BRO Beta-lactamase | penam | antibiotic target alteration |
| ASM397132v1 | 46P58B1 | 2.045 | ICR-Mc | intrinsic colistin resistant phosphoethanolamine transferase | peptide antibiotic | antibiotic target alteration |
|  |  |  | BRO-1 | BRO Beta-lactamase | penam | antibiotic inactivation |
| ASM397134v1 | 74P58B1 | 1.832 | ICR-Mc | intrinsic colistin resistant phosphoethanolamine transferase | peptide antibiotic | antibiotic target alteration |
|  |  |  | BRO-1 | BRO Beta-lactamase | penam | antibiotic inactivation |
| ASM397136v1 | 5P47B2 | 1.9 | ICR-Mc | intrinsic colistin resistant phosphoethanolamine transferase | peptide antibiotic | antibiotic target alteration |
| 49595_F01 | NCTC11020 | 1.9 | ICR-Mc | intrinsic colistin resistant phosphoethanolamine transferase | peptide antibiotic | antibiotic target alteration |
| ASM208012v1 | CCRI-195ME | 1.994 | ICR-Mc | intrinsic colistin resistant phosphoethanolamine transferase | penam | antibiotic inactivation |
| **Res-finder database** | | | | | | |
| ASM9226v1 | BBH18 | 1.863 | blaBRO-2 | amoxicillin,ampicillin,penicillin,piperacillin | peptide antibiotic | antibiotic target alteration |
| ASM74045v1 | 25240 | 1.942 | Not found | Not found | peptide antibiotic | antibiotic target alteration |
| ASM76666v1 | 25239 | 1.88 | Not found | Not found | penam | antibiotic inactivation |
| ASM207321v2 | FDAARGOS_213 | 1.908 | Not found | Not found | peptide antibiotic | antibiotic target alteration |
| ASM208012v1 | CCRI-195ME (Reference) | 1.994 | blaBRO-2 | amoxicillin,ampicillin,penicillin,piperacillin | penam | antibiotic inactivation |
| ASM298412v1 | FDAARGOS_304 | 1.942 | blaBRO-1 | amoxicillin,ampicillin,penicillin,piperacillin | peptide antibiotic | antibiotic target alteration |
| ASM397128v1 | 74P50B1 | 1.832 | Not found | Not found | penam | antibiotic target alteration |
| ASM397130v1 | 142P87B1 | 1.909 | blaBRO-1 | amoxicillin,ampicillin,penicillin,piperacillin | peptide antibiotic | antibiotic target alteration |
| ASM397132v1 | 46P58B1 | 2.045 | blaBRO-1 | amoxicillin,ampicillin,penicillin,piperacillin | penam | antibiotic inactivation |
| ASM397134v1 | 74P58B1 | 1.832 | Not Found | Not Found | peptide antibiotic | antibiotic target alteration |
| ASM397136v1 | 5P47B2 | 1.9 | Not Found | Not Found | penam | antibiotic inactivation |
| 49595_F01 | NCTC11020 | 1.9 | Not Found | Not Found | peptide antibiotic | antibiotic target alteration |
| ASM208012v1 | CCRI-195ME | 1.994 | blaBRO-2 | amoxicillin,ampicillin,penicillin,piperacillin | peptide antibiotic | antibiotic target alteration |

**Table S2: Significant Gene ontology and Molecular pathway**

| **Term ID** | **Term description** | **Observed gene count** | **Background gene count** | **Strength** | **False discovery rate** |
| --- | --- | --- | --- | --- | --- |
| **Biological Process** | | | | | |
| GO:0008152 | Metabolic process | 54 | 983 | 0.25 | 2.59E-08 |
| GO:0044237 | Cellular metabolic process | 51 | 862 | 0.28 | 2.59E-08 |
| GO:0071704 | Organic substance metabolic process | 49 | 850 | 0.27 | 4.09E-07 |
| GO:0009987 | Cellular process | 55 | 1144 | 0.19 | 1.02E-06 |
| GO:0044249 | Cellular biosynthetic process | 36 | 475 | 0.39 | 1.41E-06 |
| GO:1901576 | Organic substance biosynthetic process | 36 | 477 | 0.39 | 1.41E-06 |
| GO:0044238 | Primary metabolic process | 43 | 709 | 0.29 | 3.99E-06 |
| GO:0044281 | Small molecule metabolic process | 29 | 375 | 0.4 | 6.06E-05 |
| GO:0044271 | Cellular nitrogen compound biosynthetic process | 24 | 278 | 0.45 | 0.00015 |
| GO:0034641 | Cellular nitrogen compound metabolic process | 32 | 501 | 0.32 | 0.0005 |
| GO:1901564 | Organonitrogen compound metabolic process | 32 | 498 | 0.32 | 0.0005 |
| GO:1901566 | Organonitrogen compound biosynthetic process | 26 | 346 | 0.39 | 0.0005 |
| GO:0043603 | Cellular amide metabolic process | 16 | 144 | 0.56 | 0.00065 |
| GO:0006807 | Nitrogen compound metabolic process | 39 | 719 | 0.25 | 0.0007 |
| GO:0043604 | Amide biosynthetic process | 14 | 124 | 0.56 | 0.002 |
| GO:0034645 | Cellular macromolecule biosynthetic process | 17 | 187 | 0.47 | 0.0031 |
| GO:0019752 | Carboxylic acid metabolic process | 18 | 225 | 0.42 | 0.0077 |
| GO:0009150 | Purine ribonucleotide metabolic process | 8 | 54 | 0.68 | 0.019 |
| GO:0006730 | One-carbon metabolic process | 4 | 9 | 1.16 | 0.0205 |
| GO:0006457 | Protein folding | 5 | 21 | 0.89 | 0.0351 |
| GO:0006412 | Translation | 10 | 99 | 0.52 | 0.0455 |
| GO:0044260 | Cellular macromolecule metabolic process | 21 | 345 | 0.3 | 0.0477 |
| GO:0006099 | Tricarboxylic acid cycle | 4 | 13 | 1 | 0.0484 |
| **Molecular Function** | | | | | |
| GO:0036094 | Small molecule binding | 29 | 365 | 0.41 | 0.00016 |
| GO:0003824 | Catalytic activity | 46 | 902 | 0.22 | 0.00036 |
| GO:0097159 | Organic cyclic compound binding | 36 | 579 | 0.31 | 0.00036 |
| GO:1901363 | Heterocyclic compound binding | 36 | 579 | 0.31 | 0.00036 |
| GO:0043168 | Anion binding | 25 | 330 | 0.39 | 0.00086 |
| GO:0000166 | Nucleotide binding | 24 | 332 | 0.37 | 0.0027 |
| GO:0005488 | Binding | 41 | 815 | 0.21 | 0.0027 |
| GO:0017076 | Purine nucleotide binding | 20 | 253 | 0.41 | 0.0035 |
| GO:0043167 | Ion binding | 32 | 579 | 0.25 | 0.007 |
| GO:0035639 | Purine ribonucleoside triphosphate binding | 19 | 250 | 0.39 | 0.0079 |
| GO:0032555 | Purine ribonucleotide binding | 19 | 252 | 0.39 | 0.008 |
| GO:0097367 | Carbohydrate derivative binding | 20 | 275 | 0.37 | 0.008 |
| GO:0030554 | Adenyl nucleotide binding | 17 | 226 | 0.39 | 0.0165 |
| GO:0051082 | Unfolded protein binding | 4 | 12 | 1.03 | 0.0357 |
| GO:0005524 | ATP binding | 16 | 223 | 0.37 | 0.0372 |
| **Cellular Component** | | | | | |
| GO:0005622 | Intracellular | 53 | 819 | 0.32 | 5.40E-12 |
| GO:0005737 | Cytoplasm | 44 | 721 | 0.3 | 6.96E-07 |
| GO:0110165 | Cellular anatomical entity | 57 | 1296 | 0.16 | 6.96E-07 |
| GO:0045239 | Tricarboxylic acid cycle enzyme complex | 3 | 3 | 1.51 | 0.0155 |
| **KEGG** | | | | | |
| mct01100 | Metabolic pathways | 33 | 584 | 0.26 | 0.0056 |
| mct00020 | Citrate cycle (TCA cycle) | 5 | 20 | 0.91 | 0.0364 |
| mct03018 | RNA degradation | 5 | 21 | 0.89 | 0.0364 |
| mct01200 | Carbon metabolism | 8 | 68 | 0.58 | 0.0404 |
| mct01110 | Biosynthesis of secondary metabolites | 16 | 238 | 0.34 | 0.0466 |
| mct03010 | Ribosome | 7 | 56 | 0.61 | 0.0466 |

**Table S3: Details information for Sunburst plot**

| **Unique Gene name** | **Resistance mechanism** | **drug Class** |
| --- | --- | --- |
| blaBRO-2 | antibiotic inactivation | beta-lactam |
| ICR-Mc | antibiotic target alteration | peptide antibiotic |
| BRO-1 | antibiotic inactivation | penam |
| S10p | antibiotic target in susceptible species | Tetracyclines, Glycylcyclines |
| rpoB | antibiotic target in susceptible species | Rifamycins, Peptide antibiotics |
| S12p | antibiotic target in susceptible species | Aminoglycosides |
| gidB | gene conferring resistance via absence | Aminoglycosides |
| Iso-tRNA | antibiotic target in susceptible species | Mupirocin |
| MurA | antibiotic target in susceptible species | Fosfomycin |
| gyrA | antibiotic target in susceptible species | Fluoroquinolones Quinolones Quinolines |
| EF-G | antibiotic target in susceptible species | Fusidic acid |
| folA, Dfr | antibiotic target in susceptible species | Diaminopyrimidines |
| Rho | antibiotic target in susceptible species | Bicyclomycins |
| inhA, fabI | antibiotic target in susceptible species | Isoniazid, Ethionamide, Triclosan |
| PgsA | protein altering cell wall charge conferring antibiotic resistance | Peptide antibiotics |
| Alr | antibiotic target in susceptible species | Cycloserine |
| kasA | antibiotic target in susceptible species | Isoniazid, Triclosan |
| folP | antibiotic target in susceptible species | Sulfonamides |
| OxyR | regulator modulating expression of antibiotic resistance genes | Isoniazid |
| EF-Tu | antibiotic target in susceptible species | Elfamycins |
| Dxr | antibiotic target in susceptible species | Fosmidomycin |
| FabG | antibiotic target replacement protein | Triclosan |
| rpoC | antibiotic target in susceptible species | Myxopyronins Corallopyronins, Peptide antibiotics |
| gyrB | antibiotic target in susceptible species | Fluoroquinolones Quinolones Quinolines, Aminocoumarin antibiotics |
| Ddl | antibiotic target in susceptible species | Cycloserine |
| HtdX | antibiotic target replacement protein | No |
| fabI | antibiotic target in susceptible species | isoniazid,ethionamide,triclosan |
| ddlB | antibiotic target in susceptible species | cycloserine |
| Tuf | antibiotic target in susceptible species | elfamycins |
| Alr | antibiotic target in susceptible species | cycloserine |
| fusA | antibiotic target in susceptible species | fusidic acid |
| rpsL | antibiotic target in susceptible species | aminoglycosides |
| rpsJ | antibiotic target in susceptible species | tetracyclines,glycylcyclines |
| fabF | antibiotic target in susceptible species | isoniazid,triclosan |
| bro-2 | antibiotic inactivation enzyme | beta-lactam antibiotics |
| rplF | antibiotic target in susceptible species | fusidic acid |
| murA | antibiotic target in susceptible species | fosfomycin |
| pgsA | protein altering cell wall charge conferring antibiotic resistance | Peptide antibiotics |
| folA | antibiotic target in susceptible species | Diaminopyrimidines |
| ileS | antibiotic target in susceptible species | mupirocin |
| atpD | Drug target | No |
| nifU | Drug target | No |
| rplJ | Drug target | No |
| Mdh | antibiotic target in susceptible species | No |
| fghA | antibiotic target replacement protein | No |
| msrB | antibiotic target in susceptible species | No |
| purM | antibiotic target in susceptible species | No |
| thyA | antibiotic target in susceptible species | No |
| nuoB | antibiotic target in susceptible species | No |
| metK | antibiotic target in susceptible species | No |
| purD | antibiotic target in susceptible species | No |
| fumC | antibiotic target in susceptible species | No |
| gale | antibiotic target in susceptible species | No |
| iscS | antibiotic target in susceptible species | No |
| rocD | antibiotic inactivation | No |
| Eno | antibiotic target alteration | No |
| argG | antibiotic inactivation | No |
| atpA | antibiotic inactivation | No |
| sucB | antibiotic target alteration | No |
| Fba | Drug target | No |
| groL | Drug target | No |
| ahcY | Drug target | No |
| Ung | antibiotic target in susceptible species | No |
| clpP | antibiotic target replacement protein | No |
| folE | antibiotic target in susceptible species | No |
| dnaK | antibiotic target in susceptible species | No |
| blaBRO-1 | antibiotic target in susceptible species | No |
| kata | Stress protein | No |
| hktE | Stress protein | No |
| OxyR | regulator modulating expression of antibiotic resistance genes | Isoniazid |
| rsmG | gene conferring resistance via absence | aminoglycosides |
| fabG_1 | antibiotic target replacement protein | triclosan |
| tufA_2 | antibiotic target in susceptible species | elfamycins |
| tufA_1 | antibiotic target in susceptible species | elfamycins |
| eptA | protein altering cell wall charge conferring antibiotic resistance | polymixins |
| dfrA | antibiotic target in susceptible species | diaminopyrimidines |
